# A breathing mask attenuates acute airway responses to exercise in sub-zero environment in healthy subjects

**DOI:** 10.1007/s00421-022-04939-x

**Published:** 2022-04-07

**Authors:** Nikolai Stenfors, Hampus Persson, Alasdair Tutt, Ellen Tufvesson, Erik P. Andersson, Mats Ainegren, Helen G. Hanstock

**Affiliations:** 1 Unit of Medicine, Department of Public Health and Clinical Medicine, Umeå University, Umeå, Sweden; 2 Swedish Winter Sports Research Centre, Department of Health Sciences, Mid Sweden University, Östersund, Sweden; 3 Department of Clinical Sciences Lund, Respiratory Medicine and Allergology, Lund University, Lund, Sweden; 4 Sports Tech Research Centre, Department of Quality Management and Mechanical Engineering, Mid Sweden University, Östersund, Sweden

**Keywords:** Heat- and moisture-exchanging breathing device, Airway epithelial damage, Cold temperature, Exercise-induced bronchoconstriction

## Abstract

**Purpose:**

Cold air exposure is associated with increased respiratory morbidity and mortality. Repeated inhalation of cold and dry air is considered the cause of the high prevalence of asthma among winter endurance athletes. This study assessed whether a heat- and moisture-exchanging breathing device (HME) attenuates airway responses to high-intensity exercise in sub-zero temperatures among healthy subjects.

**Methods:**

Using a randomized cross-over design, 23 healthy trained participants performed a 30-min warm-up followed by a 4-min maximal, self-paced running time trial in − 15 °C, with and without HME. Lung function was assessed pre- and immediately post-trials. Club cell protein (CC-16), 8-isoprostane, and cytokine concentrations were measured in plasma and urine pre- and 60 min post trials. Symptoms were assessed prior to, during, and immediately after each trial in the chamber.

**Results:**

HME use attenuated the decrease in forced expiratory volume in 1 s (FEV_1_) post trials (∆FEV_1_: mean (SD) HME − 0.5 (1.9) % vs. no-HME − 2.7 (2.7) %, *p* = 0.002). HME also substantially attenuated the median relative increase in plasma-CC16 concentrations (with HME + 27% (interquartile range 9–38) vs no-HME + 121% (55–162), *p* < 0.001) and reduced airway and general symptom intensity, compared to the trial without HME. No significant changes between trials were detected in urine CC16, 8-isoprostane, or cytokine concentrations.

**Conclusion:**

The HME attenuated acute airway responses induced by moderate-to-maximal-intensity exercise in − 15 °C in healthy subjects. Further studies are needed to examine whether this HMEs could constitute primary prevention against asthma in winter endurance athletes.

**Supplementary Information:**

The online version contains supplementary material available at 10.1007/s00421-022-04939-x.

## Introduction

Exposure to cold air is associated with increased morbidity and mortality in all age groups of our population (Rocklov and Forsberg 2008). Especially sensitive are the elderly and patients with cardiopulmonary disease (Schwartz 2005). Children undertaking physical activity in cold temperatures commonly report respiratory symptoms (Rasi et al. 2017).

Repeated and prolonged inhalation of cold air is believed to be the cause of the high prevalence of exercise-induced bronchoconstriction (EIB) and asthma in winter endurance athletes such as cross-country skiers (Kippelen et al. 2018; Mäki-Heikkilä et al. 2020). Inhalation of cold air leads to drying of the airways, accelerating water loss from the lower airways that may increase intracellular osmolarity in the airway cells. This may lead to cell shrinkage with the release of inflammatory and broncho-constrictive mediators, such as leukotrienes and prostaglandins (Kippelen et al. 2018).

Further proposed mechanisms in the pathogenesis of EIB in athletes are airway epithelial injury, airway inflammation, and oxidative stress (Atchley and Smith 2020). Exercise in cold, dry air has been shown to increase urine CC16 concentrations to a greater extent than exercise in warm, humid air (Bolger et al. 2011), indicating increased airway epithelial damage in colder climates. Endurance athletes also show increased airway inflammation (Bonsignore et al. 2003), even though cold exposure per se has limited influence on systemic immune function. An exercise challenge has been shown to increase the levels of the type 2 inflammatory biomarker interleukin-13 in plasma (Tufvesson et al. 2020). Competitive swimmers, another population with an increased risk of “sports asthma” (Couto et al. 2015), show signs of increased airway oxidative stress (Morissette et al. 2016). It is unclear whether oxidative stress and systemic inflammation arise as a result of strenuous exercise in cold conditions.

As cold climates detrimentally affect both public health and populations who habitually undertake physical activity in cold environments, preventive interventions are needed. A heat- and moisture-exchanging breathing device (HME) has capacity to warm and humidify inhaled air and it is well known that HMEs attenuate EIB in sub-zero temperatures in subjects with asthma (Nisar et al. 1992; Millqvist et al. 1995; Beuther and Martin 2006). Recently, this was also shown in healthy subjects (Frischhut et al. 2020).

If HMEs attenuate cold-air-induced acute airway responses in healthy subjects, they could possibly function as a primary prevention method against asthma induced by endurance training in sub-zero environments, for example in cross-country skiers. Furthermore, they could limit the cold air-associated morbidity in a general population. Our study aims were therefore to investigate whether HMEs in healthy subjects attenuate airway responses induced by strenuous physical activity in sub-zero air. Our primary hypothesis was that HME use would attenuate a decrease in forced expiratory volume in 1 s (FEV_1_). Exploratory hypotheses were to examine whether HMEs attenuate any biochemical signs of airway injury, oxidative stress, airway and systemic inflammation, as well as respiratory symptoms.

## Methods

### Participants

A total of 23 healthy (8 women), trained adults, mean age 31 (range 18–53 years), completed the study. Participants were recreational or competitive, bordering elite athletes across a range of endurance-trained backgrounds and all were accustomed to performing endurance training in sub-zero temperatures. Exclusion criteria included current use of prescription medication (except hormonal contraceptives), recent respiratory illness, physician-diagnosed asthma, or symptoms of allergies to common airborne allergens. A description of the study population is presented in Table 1. An extensive description of the same study population has been published (Tutt et al. 2021). Participants were requested to not perform any arduous training in the 48 h preceding each trial, avoid caffeine on the day of each trial, and alcohol for 24 h beforehand (Hallstrand et al. 2018). Furthermore, before participants were asked to arrive well hydrated and avoid exercise, such as running, brisk walking, and bicycling, on their way to the lab. Clothing was recommended as a lightweight hat/headband, a fabric neck warmer, gloves, base layer, light windproof jacket, and tights/pants. The neck warmer was not allowed to cover the face. All participants gave written informed consent, and the study was approved by the Umeå Regional Ethical Review Board (Dnr: 2018–419-31).

**Table 1:** A description of the study population of 15 men and 8 women

Age, years	31 (8)
Height, cm	176 (9)
Weight, kg	71 (11)
VO_2peak,_ ml^.^kg^−1.^min^−1^	54 (7)
VE_peak,_ L^.^min^−1^	146 (32)
FEV_1_, % of predicted	105 (10)
FVC, % of predicted	104 (10)
FEV_1_/FVC, % of predicted	101 (5)
R5Hz, % of predicted	106 (25)
R20Hz, % of predicted	122 (29)

Mean (SD)*VO*_*2peak*_ peak oxygen uptake, *VE*_*peak*_ peak ventilation, *FEV*_*1*_ forced expiratory volume in 1 s, *FVC* forced vital capacity, *R5Hz and R20Hz* airway resistance at 5 and 20 Hertz

### Study design

Participants performed a familiarization test followed by two experimental trials with and without the HME in a randomized cross-over design. Participants used an Airtrim mask (Vapro AB, Västerås, Sweden) with a Sport filter during the HME trial. The Airtrim mask was chosen as it is the most used HME among Swedish adolescent skiers (Stenfors et al. 2020) and provides minimal resistance to breathing (Ainegren et al. 2020).

The study was conducted in May and June 2019 in an environmental chamber involving treadmill running with the capability for speed to be self-controlled by the participant (Rodby 2700E, Rodby Innovation AB, Vänge, Sweden). The environmental chamber has previously been described in detail (Sjöström et al. 2019). The chamber has a temperature but not a humidity controller. Temperature, relative humidity, and absolute humidity in the chamber were (mean ± SD) − 14.9 ± 0.2 °C, 69.5 ± 5.1%, and 1.3 ± 0.1 g·m^−3^ during the HME trials, and − 15.0 ± 0.1 °C and 70.2 ± 4.1%, and 1.3 ± 0.1 g·m^−3^ during the trials without the HME (no-HME). Trials were conducted at the same time of day, and at least 72 h apart to avoid crossover effects. The mean (min–max) outdoor temperature during the trial days was 11 (4–18) °C.

### Familiarization test

Before the first experimental trial, participants performed a familiarization running protocol at − 5 °C in the environmental chamber, at a gradient of 4%, while wearing a portable breath-by-breath metabolic cart system (Metamax 3B, Cortex Biophysik, Leipzig, Germany). The familiarization protocol consisted of three 5-min submaximal stages, a 15-min self-selected warm-up, and finally, a 4-min self-paced treadmill running time trial (TT) to determine peak oxygen uptake (VO_2peak_). Regression analysis of speed and submaximal oxygen uptake (VO_2_) together with measured VO_2peak_ was used to revise submaximal speeds ahead of the main experimental trials. The familiarization test has been described in detail previously (Tutt et al. 2021).

### Experimental protocol

During the experimental trials, participants performed impulse oscillometry (IOS) and dynamic spirometry, provided blood and urine samples, and reported respiratory symptoms before and after exercise in the environmental chamber. The exercise protocol has been described previously (Tutt et al. 2021) and was designed to simulate a thorough warm-up followed by a 4-min maximal effort, i.e., similar duration and intensity to a cross-country sprint skiing competition. Athletes and coaches report that sprint skiing competitions are known to often induce acute post-exercise cough and airway irritation, symptoms that in some cases may last for several hours. Indeed, “sprint cough” is a term commonly used by competitive skiers. Participants performed a 30-min graded submaximal warm-up at fixed speeds to elicit 65–90% VO_2peak_ (0–5 min: 65% VO_2peak_; 5–10 min: 70% VO_2peak_; 10–15 min: 75% VO_2peak_; 15–18 min: 90% VO_2peak_; 18–30 min: 65% VO_2peak_). After a 5-min rest inside the chamber, participants then performed a 4-min maximal effort TT. As previously reported (Tutt et al. 2021), mean distance covered during the TT was 13 m less with HME than without (918 + 110 vs. 931 + 106 m), but physiological demands were similar or higher with HME. Participants recovered in the chamber for 5 min after the conclusion of the TT. During the 5-min rest periods, the participants filled out questionnaires and adjusted clothing if needed.

### Lung function

Impulse oscillometry (IOS) followed by dynamic spirometry (Jaeger Vyntus IOS, CareFusion, Germany) were performed according to international guidelines (Graham et al. 2019; King et al. 2020) and before entering the chamber. IOS was repeated immediately after exiting the chamber, 5 min after the TT. For IOS, the main outcome measures were resistance at 5 Hz (R5Hz), resistance at 20 Hz (R20Hz), reactance at 5 Hz (X5Hz), and resonant frequency (F_res_). Dynamic spirometry was repeated immediately after IOS, 10 min after the TT. Main outcome measures were forced expiratory volume in 1 s (FEV_1_), forced vital capacity (FVC), and forced expiratory flow at 25%, 50%, and 75% of FVC (FEF25, FEF50, FEF75).

### Blood and urine sampling

Venous blood and urine samples were collected pre- and 60 min post chamber exit. Blood samples were obtained via venipuncture of an antecubital vein into two 3 ml EDTA vacuum tubes. Sixty-minute post-exposure was chosen to detect peak plasma and urine concentrations of CC16 (Tufvesson et al. 2013). The sample used for cytokine analysis was immediately placed on ice. Samples were immediately centrifuged at 2130×*g* at 20 °C for 10 min for CC16 and 8-isoprostane analyses and 1300×*g* at 4 °C for cytokine analyses.

Participants were instructed to avoid going to the toilet in the hours before the first urine sample was collected. Male participants were instructed to discharge the first 100 ml of each urine sample to eliminate the post-renal excretion of club cell protein 16 (CC16) from the prostate (Andersson et al. 2007).

Plasma and urine samples were aliquoted and stored at − 80 °C prior to analysis. Prior to storage, 10 µl 0.05% butylated hydroxy-toluene was added per 1 ml for aliquots subsequently analyzed for 8-isoprostane, to prevent oxidation of samples during storage.

### CC16 and 8-isoprostane

CC16 and 8-isoprostane were analyzed in plasma and urine using commercially available ELISA kits (Human Clara Cell Protein ELISA kit, Biovendor, Modrice, Czech Republic; 8-isoprostane ELISA kit, Cayman Chemical, Ann Arbor, MI, USA). Urine-CC16 and 8-isoprostane were corrected for creatinine to adjust for different dilutions. Mean intra-assay coefficients of variation were < 5% for all plates.

### Cytokine analyses

Plasma samples were analyzed for a panel of ten cytokines (IL-1β, IL-4, IL-5, IL-6, IL-8, IL-10, IL-13, IL-25 (17E), GM-CSF, and TNF-α) using a multiplex immunoassay (U-plex, Meso Scale Diagnostics, Rockville, MD). For IL-10, IL-6, IL-8, and TNF-ɑ, all samples were within the range of the assay standard curve. For IL-5, 85% samples were within the range of the assay standard curve, and concentrations could be obtained for all remaining samples through minor extrapolation of the standard curve. For GM-CSF, IL-13, IL-25, IL-1β, and IL-4, only 18%, 13%, 15%, 30%, and 20% of samples had detectable concentrations within the assay range; out of range samples all had lower concentrations than the lower limit of detection (Supplementary Table 1).

### Symptoms

Two questionnaires were administered at four time points: immediately pre-trial outside the chamber (pre), between the warm-up and TT (warm-up), immediately after the TT, and after exiting the chamber, impulse oscillometry, and spirometry (post).

The first questionnaire surveyed airway and general symptoms common during exercise in cold air (Sjöström et al. 2019). Participants used Borg CR10-scales to rate the intensity of the symptoms, where 0 represented “no symptoms” and 10 represented “extremely severe symptoms”. Symptoms after warm-up, TT, and post trials were added together into a sum-score for each subject.

At similar time points, participants assessed the thermal environment using the ISO 10551:1995 questionnaire (Institute 2001). Perceptual judgment of personal thermal state using the question “How are you feeling now?” had seven levels ranging from “Very hot” to “Very cold”. An evaluative judgment on personal thermal state using the question “Do you find this…” had five levels ranging from “Comfortable” to “Extremely uncomfortable”. Their thermal preference was assessed using the question “Please state how would you prefer to be now” and a 7-degree scale from “Much warmer” to “Much cooler”? Personal tolerance to the thermal conditions was assessed by “Is it…?” and a 5-degree scale from “Perfectly tolerable” to “Intolerable”.

### Statistical analyses

A sample size calculation was conducted using ΔFEV_1_ as the primary outcome variable. We assumed a mean of FEV_1_ = 4.58 L, SD = 0.40, and that exercise in − 15 °C without HME would decrease FEV_1_ by 6% compared to with HME (Therminarias et al. 1998; Kennedy and Faulhaber 2018). We assumed equal variance and a correlation of 0.3 between trials. With an alpha of 0.05 and a power of 0.80, 20 participants were needed.

Change in lung function and concentrations of CC16 and 8-isoprostane in plasma and urine were compared within trials (post – pre) and between trials (change with HME vs change without HME) using Wilcoxon signed rank-sum tests or paired t tests, depending on whether each variable was judged, by visual inspection and Shapiro–Wilk test, to be normally or non-normally distributed. Wilcoxon signed rank-sum tests were also used to determine differences in cytokine concentrations within and between trials for cytokines with > 75% samples within the detection range. Changes in 8-isoprostane concentrations within and between trials were compared using the sign test, as the differences were neither normally distributed nor symmetrical.

For the cytokines with < 75% samples in range, data were transformed into dichotomous categories (above versus below the lower detection limit (LDL)) and analyzed using one-sample proportion tests. As no samples had cytokine concentrations above the upper detection limit, this approach allowed analysis of whether the proportion of samples with cytokine concentrations below detection limits of the assay differed within and between trials.

Symptoms and thermal assessment data were not normally distributed. Symptoms were compared between trials at all four time points as well as the sum scores using the Wilcoxon signed rank-test on paired samples. For statistical analyses, the scales assessing thermal stress were converted to numerical values (Institute 2001). A *p* value < 0.05 was considered statistically significant.

## Results

**Fig. 1:**
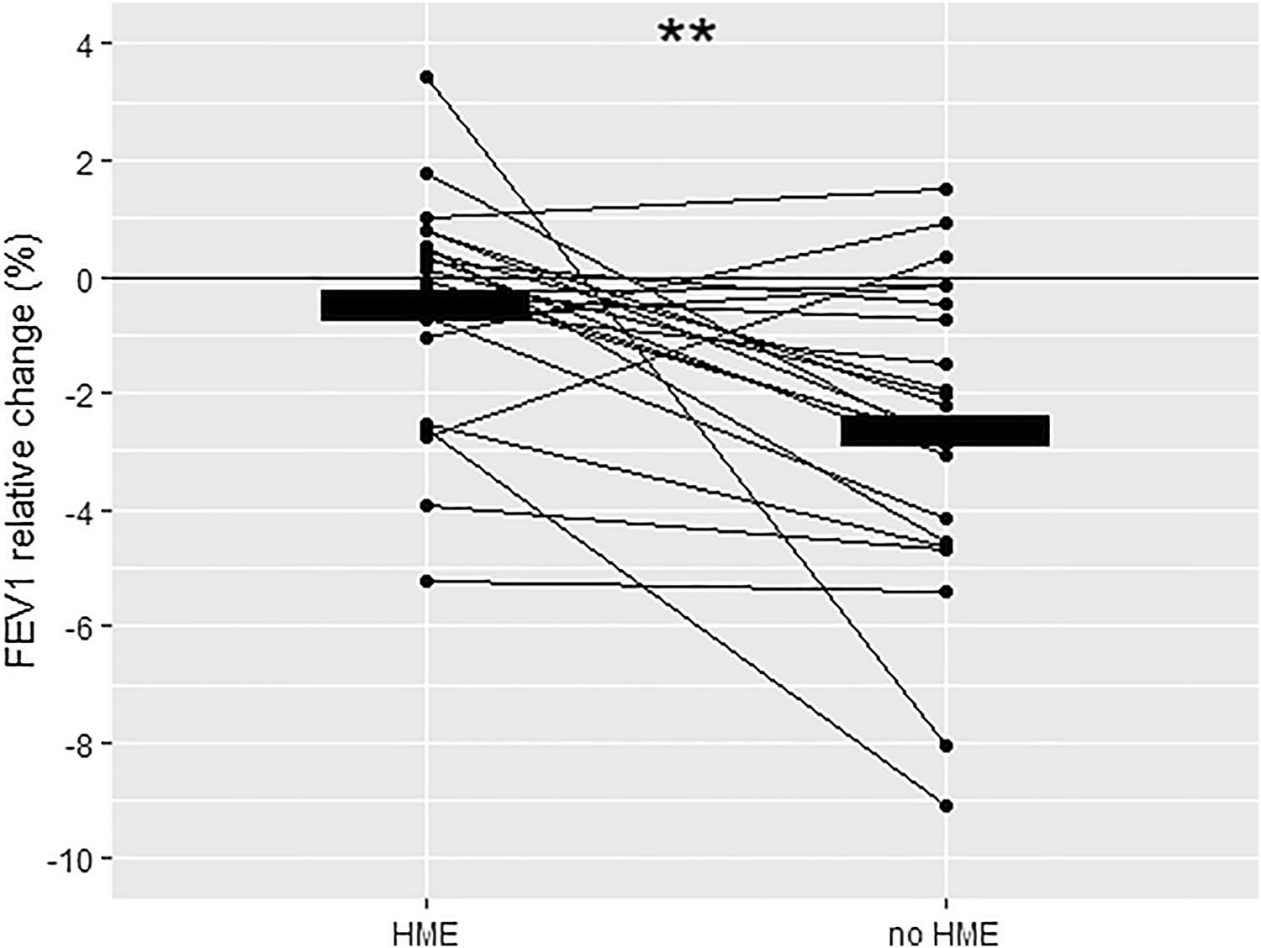
Individual relative ((post–pre)/pre) changes in FEV_1_ for each trial. Bold horizontal lines illustrate group means. Significant changes (post–pre) between trials (*p* = 0.002)

Lung function responses within and between trials are presented in Table 2. Relative reductions of FEV_1_ within trials are illustrated in Fig. 1. None of the subjects experienced clinically significant EIB, as defined by a relative reduction of FEV_1_ ≥ 10%. HME use during the warm-up and time trial completely attenuated the significant decreases in FEV_1_ (*p* = 0.002) and FEV25 (*p* = 0.016) observed after the trial without HME. No decrement in FEF50 was observed in the HME trial, whereas a significant decrease in FEF50 was observed without the HME (*p* = 0.021). In both trials, a significant decrease in FVC occurred, of similar volume. Impulse oscillometry did not detect any statistically significant responses within or between trials.

**Table 2:** Spirometry and IOS performed by the subjects before (pre) and after (post) exercise with and without HME

	HME			No HME			
	Pre	Post	*p* value*	Pre	Post	*p* value*	*p* value^†^
FEV_1_, L	4.48 (0.89)	4.46 (0.89)	0.235	4.47 (0.86)	4.35 (0.81)	** < 0.001**	**0.002**
FVC, L	5.54 (1.12)	5.45 (1.14)	**0.005**	5.55 (1.11)	5.43 (1.11)	**0.001**	0.268
FEF25, L^.^s^−1^	8.72 (1.74)	8.52 (1.76)	0.166	8.57 (1.86)	7.89 (1.68)	** < 0.001**	**0.016**
FEF50, L^.^s^−1^	5.05 (0.98)	4.98 (1.18)	0.583	5.07 (1.14)	4.84 (1.04)	**0.021**	0.338
FEF75, L^.^s^−1^	1.94 (0.61)	2.06 (0.68)	0.064	1.94 (0.63)	1.93 (1.68)	0.911	0.071
R5Hz, kPa/(L^.^s^−1^)	0.309 (0.093)	0.316 (0.112)	0.509	0.312 (0.099)	0.319 (0.101)	0.717	0.991
R20Hz, kPa/(L^.^s^−1^)	0.306 (0.090)	0.303 (0.104)	0.750	0.309 (0.099)	0.299 (0.812)	0.424	0.714
X5Hz, kPa/(L^.^s^−1^)	− 0.072 (0.29)	− 0.071 (0.025)	0.862	− 0.073 (0.027)	− 0.073 (0.023)	0.943	0.927
Fres, Hz	9.638 (2.113)	9.906 (1.809)	0.272	9.987 (2.722)	10.347 (1.669)	0.427	0.832

Mean (SD). Significant * p* values in bold

*HME* heat- and moisture-exchanging breathing mask, *FEV*_*1*_ forced expiratory volume in 1 s, *FVC* forced vital capacity, *FEF*_*25*_*, FEF*_*50*_*, FEF*_*75*_ forced expiratory flow at 25%, 50% and 75% respectively, *R*_*5*_* Hz* resistance at 5 Hz, *R*_*20*_* Hz* resistance at 20 Hz, *X*_*5*_* Hz* reactance at 5 Hz, *F*_*res*_ resonance, *L* liters, *s* second, *kPa* kilopascal, *Hz* Hertz

^*^p value calculated for changes within trials (pre vs post)

^†^*p* value calculated for changes between trials (change with HME vs change without HME)

### CC16

**Fig. 2:**
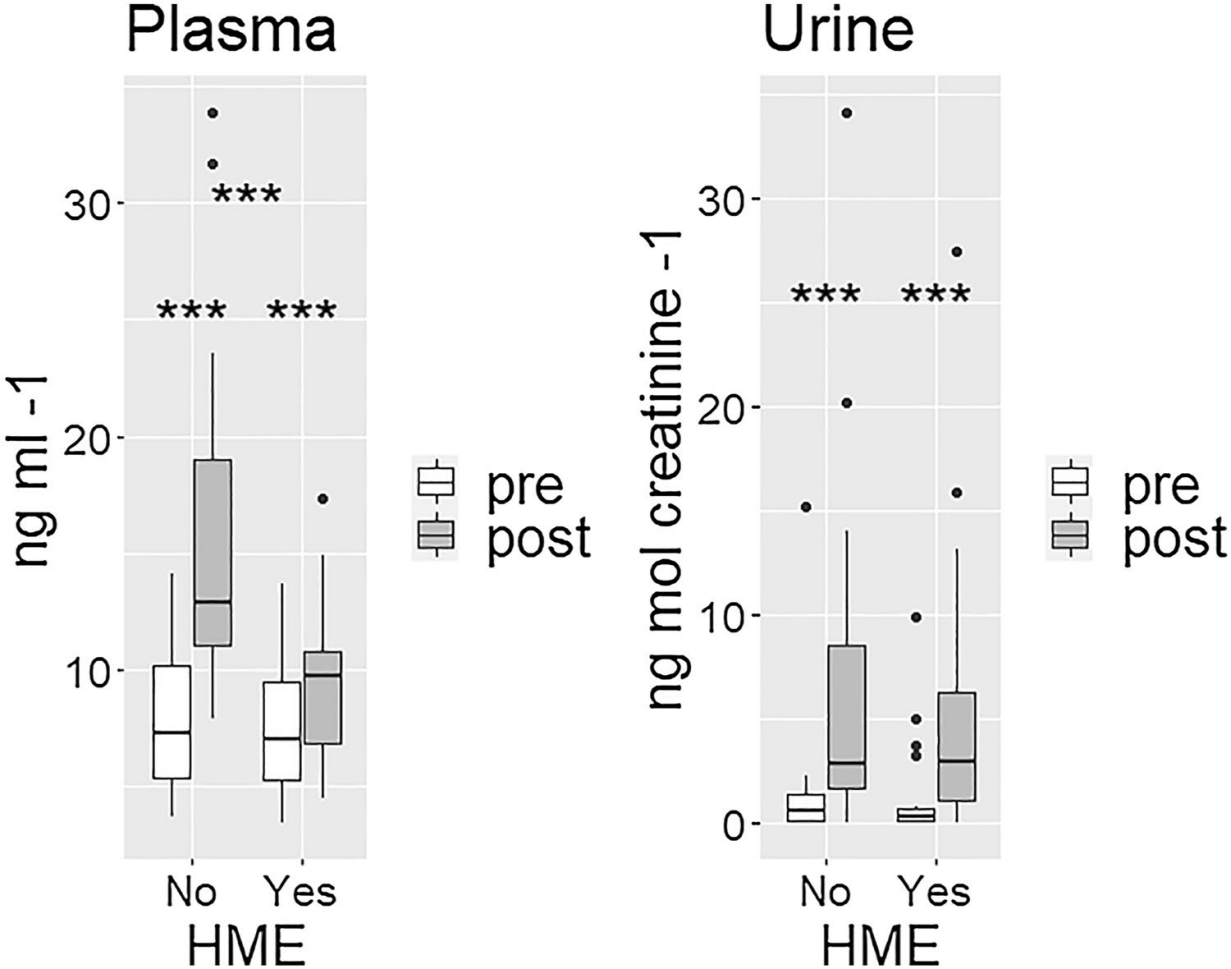
Boxplots for CC16 concentrations each pre- and post each trial. Plasma-CC16 (left figure) increased after both trials (*p* < 0.001 for both), the increase was less using a HME (*p* < 0.001). Urine-CC16 (right figure) also increased after both trials (*p* < 0.001 for both), without significant difference between trials

CC16 concentrations pre-trials did not differ in plasma (*p* = 0.26) or urine (*p* = 0.39). HME use attenuated the relative increase in plasma-CC16 concentrations observed in both trials; with HME + 27% (interquartile range 9–38) vs no-HME + 121% (55–162), *p* < 0.001. Urine-CC16 increased significantly within each trial, without significant difference between trials (Fig. 2).

### Cytokines

IL-6, IL-8, and IL-10 increased following exercise in the cold environment in both trials (*p* < 0.01, Table 3). The use of an HME did not affect IL-5, IL-6, IL-8, IL-10, and TNF-α responses to exercise in cold (Table 3). For the cytokines with the majority of samples below the detection range of the assay (GM-CSF, IL-13, IL-25, IL-1β, and IL-4), there were no significant differences in the frequency of samples in range within or between trials (Supplementary Table 1).

**Table 3:** Cytokine and 8-isoprostane concentrations in plasma and urine pre- and post-HME vs no-HME trials

	HME			No HME			
	Pre	Post	*p* value*	Pre	Post	*p* value*	*p* value†
Plasma- IL-5, pg ml^−1^	0.46 (0.35–0.61)	0.45 (0.29–0.72)	0.179	0.42 (0.27–0.59)	0.41 (0.32–0.47)	0.235	0.377
Plasma- IL-6, pg ml^−1^	0.53 (0.34–1.18)	1.10 (0.58–1.43)	** < 0.001**	0.44 (0.37–0.98)	0.95 (0.63–1.62)	** < 0.001**	0.823
Plasma- IL-8, pg ml^−1^	2.58 (2.09–3.33)	3.19 (2.54–4.10)	** < 0.001**	2.61 (2.13–2.94)	3.33 (2.60–4.08)	** < 0.001**	0.286
Plasma- IL-10, pg ml^−1^	0.33 (0.23–0.43)	0.35 (0.22–0.84)	**0.006**	0.33 (0.22–0.62)	0.38 (0.27–0.73)	**0.008**	0.142
Plasma- TNF-alpha, pg ml^−1^	1.66 (1.20–2.22)	1.62 (1.07–2.39)	0.643	1.65 (1.18–2.04)	1.46 (1.27–1.96)	0.211	0.601
Plasma-8-isoprostane, pg ml^−1^	17.4 (13.1–20.1)	18.2 (13.4–21.0)	0.678	16.5 (12.9–19.9)	16.9 (14.7–19.6)	0.210	1.00
Urine- 8-isoprostane, pg nmol creatinine ^−1^	86.4 (69.3–112.0)	78.8 (63.6–90.8)	0.134	83.4 (66.4–109.0)	73.0 (61.4–85.0)	**0.003**	0.832

Data presented as medians (interquartile ranges) and significant P values presented in bold

^*^p value calculated for changes within trials (pre vs post)

^†^*p* value calculated for changes between trials (change with HME vs change without HME)

### 8-isoprostane

Urine 8-isoprostane concentration decreased following exercise without HME in the cold environment (*p* = 0.003). No other significant changes were detected within or between trials (Table 3).

### Symptoms

**Fig. 3:**
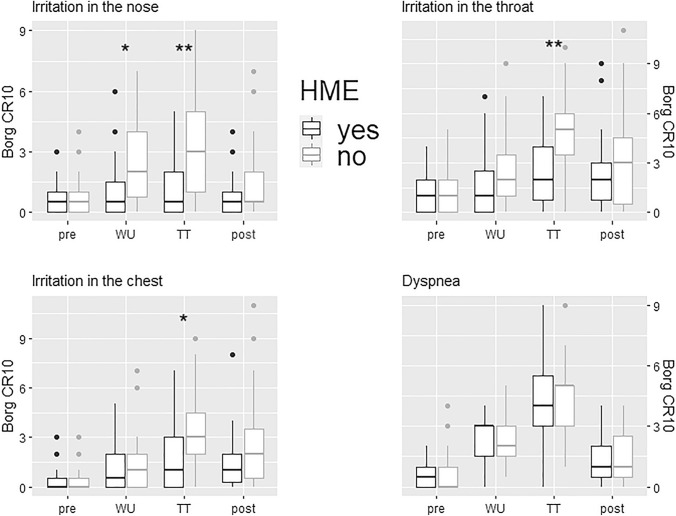
Boxplots for symptom intensity pre-, after warm-up (WU), after time trial (TT), and post each trial. HME usage attenuated irritation in the nose during warm-up (*p* = 0.012) and TT (*p* = 0.003). HME use attenuated irritation in the throat during the TT (*p* = 0.002). HME use attenuated irritation in the throat during the TT (*p* = 0.030). The level of dyspnea pre-, after warm-up, after the TT and post did not differ between trials with vs without HME

Symptoms at baseline did not differ between trials. HME use significantly reduced irritation in the upper and lower airways during warm-up and TT compared to the no-HME trial (Fig. 3). HME use did not affect dyspnea during the trials (Fig. 3). A description and comparison of other symptoms with and without HME are presented in Table 4.

**Table 4:** A comparison of symptom intensity between trials with and without HME

	HME	No HME	*p* value
Mucus in the nose			
Pre	0.5 (0.25–1.0)	0.5 (0.0–1.0)	0.524
Warm-up	3.0 (1.5–5.0)	4.0 (2.5–6-5)	0.259
Time trial	4.0 (2.5–5.5)	4.0 (3.5–7.5)	0.279
Post	0.5 (0.5–2.0)	1.0 (0.5–2.5)	0.089
Sum	9.0 (4.75–12.5)	10.0 (6.75–16.0)	0.253
Cold face			
Pre	0.0 (0.0–0.0)	0.0 (0.0–0.0)	0.071
Warm-up	2.0 (0.25–3.0)	4.0 (2.5–5.0)	** < 0.001**
Time trial	1.0 (0.75–2.5)	4.0 (1.25–6.0)	**0.010**
Post	0.5 (0.0–2.0)	0.0 (0.5–1.5)	0.843
Sum	4.0 (1.0–6.5)	9.0 (5.5–12.0)	**0.003**
Cold extremities			
Pre	0.0 (0.0–0.5)	0.0 (0.0–0.25)	0.934
Warm-up	2.0 (0.75–3.5)	3.0 (0.75–4.5)	0.697
Time trial	2.0 (1.0–3.0)	4.0 (1.5–5.0)	0.215
Post	0.5 (0.0–2.0)	1.0 (0.0–3.0)	0.606
Sum	4.0 (2.5–9.0)	7.0 (3.25–13.0)	0.480
Discomfort			
Pre	0.0 (0.0–0.0)	0.0 (0.0–1.0)	0.149
Warm-up	3.0 (1.5–4.0)	4.0 (1.5–5.0)	0.471
Time trial	2.0 (0.5–3.0)	3.0 (1.0–5.0)	0.175
Post	0.5 (0.0–1.0)	1.0 (0.0–1.5)	0.598
Sum	5.5 (2.5–8.5)	8.0 (3.75–10.00)	0.182
Warmth			
Pre	2.0 (1.5–3.0)	2.0 (0.5–3.0)	0.400
Warm-up	3.0 (2.0–4.4)	3.0 (2.0–4.0)	0.632
Time trial	4.0 (3.0–5.0)	4.0 (2.0–5.0)	0.600
Post	4.0 (2.5–4.5)	4.0 (2.5–6.5)	0.299
Sum	10.0 (7.5–14.0)	10.0 (6.5–15.0)	0.982

Data presented as medians (interquartile ranges) and significant *p* values presented in bold

Sum = warm-up + time trial + post

HME use also affected the perceptual judgment of thermal state immediately after the TT. With HME, the median feeling was “neutral”, whereas without HME, the participants felt “slightly cool” (*p* = 0.033 between trials). No other differences between trials were detected in participant assessment of thermal conditions.

## Discussion

This experimental study evaluated whether a HME protects healthy subjects against acute airway damage induced by exercise at − 15 °C. HME use during a 30-min warm-up followed by a 4-min maximal time trial was shown to: (1) diminish signs of exercise-induced bronchoconstriction, (2) reduce biochemical signs of damage to airway epithelial integrity, and (3) attenuate airway-specific symptoms as well as general symptoms, compared to the trial without HME. Acute airway damage induced by a warm-up and brief maximal exercise test in a very cold environment is therefore likely to be attenuated by HME use. The outcomes encourage us to hope that HME use could limit airway morbidity induced by cold air at a population level and function as a primary prevention strategy for airway morbidity, such as EIB and asthma, among winter endurance athletes. HME use should therefore be recommended to athletes training in a very cold environment, i.e., at temperatures similar, or colder, to the − 15 °C that was used in the current study.

### HMEs attenuate signs of exercise-induced bronchoconstriction

The brief but maximal physical exertion in − 15 °C did not induce clinically significant decreases in FEV_1_ in the participants. Despite this, HME still provided the healthy volunteers excellent protection against the small decrements in FEV_1,_ i.e., discreet signs of subclinical exercise-induced bronchoconstriction. This is supported by our present findings as well as a recent study that utilized an 8-min high-intensity treadmill exercise test at − 20 °C (Frischhut et al. 2020). The recent study found that an HME protected against small decrements in FEV_1_, FEF25, FEF50, and FEF75, while we detected that HME use only attenuated decrements in FEV_1_ and FEF25. Changes in FEV25 to FEF75 are difficult to interpret, as they are sensitive to changes in FVC, which occurred in both our trials. Taken together, HMEs appear to mostly protect against proximal airway obstruction, where airway drying is probably greatest. Based on this assumption, we are slightly surprised that we did not detect any increases in airway resistance in the proximal airways, as measured by R20Hz. This result was likely due to the large variability in R20Hz values and thus, for the detection of a significant change, a larger sample size would probably have been required.

FVC was significantly reduced, by similar magnitudes, in both trials. FVC reductions post exercise have been observed in − 20 °C (Frischhut et al. 2020) and − 11 °C (Chapman et al. 1990). In contrast to Frischhut et al. (Frischhut et al. 2020), we did not detect that HMEs protect against decrements in FVC. Possible mechanisms of exercise-induced reductions in FVC include poor effort due to respiratory muscle fatigue (Mahler and Loke 1981), extra-pulmonary restriction by the cold environment, and increased residual volume after exercise due to increased pulmonary blood flow leading to reduced pulmonary compliance (Stubbing et al. 1980). Irrespective of the triggers leading to the small decrease in FVC after exercise in sub-zero temperatures, HME use did not prevent these mechanisms.

The small decrease in FEV_1_ observed in the present trial was probably triggered by cold and dry air, which could be estimated to reach around 25 °C at the bronchial level (McFadden et al. 1985), in the tracheobronchial tree. However, another trigger for the mild bronchoconstriction could have been facial cooling leading to stimulation of the trigeminal nerve and subsequent constriction of bronchial smooth muscle (Josenhans et al. 1969; Berk et al. 1987; Koskela and Tukiainen 1995; Koskela et al. 1996). The suggested pathogenesis of this cold- and dry air-induced bronchoconstriction in the healthy subjects is a hyperosmotic shift of the extracellular fluid in the airway mucosa. This would then via airway epithelial injury directly stimulate of afferent C-fiber nerve endings and immediate local release of neuropeptides or stimulate cell shrinkage and release of mediators that trigger bronchial smooth muscle contraction, vascular leakage, and edema (Atchley and Smith 2020). The HME might have attenuated this response by increasing the temperature and humidity of the inhaled air and protecting the lower facial area and thus facial cooling, thus reducing both potential triggers.

### HMEs attenuate airway epithelial damage induced by exercise in sub-zero air

HME use substantially attenuated the increase in plasma-CC16 concentrations induced by the trial. We suggest that moderate-to-maximal intensity, medium-duration exercise in − 15 °C causes epithelial injury, provoking the airway sub-epithelium and parasympathetic afferent C-fiber nerve endings. We imply that HME usage protects against this cold- and dry air-induced airway epithelial injury by warming and humidifying inhaled cold, dry air. Non-ciliated club cells in respiratory bronchioles, and in lesser numbers in terminal bronchioles, secrete the protein CC16. CC16 is considered a biomarker for airway epithelial damage, even though its usefulness has been questioned (Lakind et al. 2007). Epithelial damage results in enhanced permeability of the airway epithelia to CC16, causing CC16 to leak into other fluid compartments and increases blood CC16 concentration (Hermans and Bernard 1999). Whereas blood samples represent a “snap-shot” of the CC16 concentrations, urine samples are dependent on glomerular filtration and mirror an accumulation of CC16. Signs of exercise-induced epithelial injury have previously been detected after swimming (Romberg et al. 2011).

### HMEs did not affect cytokine responses or 8-isoprostane

Airway and systemic inflammation and oxidative stress could indicate that the strain put upon the airways has overwhelmed their protective capacity. The acute increase in systemic IL-6 observed in both trials was likely a consequence of increased production in skeletal muscle in response to exercise; the magnitude of post-exercise IL-6 responses is understood to be proportional to exercise intensity, duration and active muscle mass (Fischer 2006). IL-6 is thought to be involved in exercise-related metabolic changes but like IL-10, primarily as an anti-inflammatory myokine (Pedersen and Febbraio 2008). The pro-inflammatory and neutrophil chemotactic factor IL-8 increased in both trials. IL-8 can be released from working skeletal muscles but also by bronchial epithelial cells, fibroblasts, and airway smooth muscle cells. Increased concentrations of IL-8 in sputum have been found in competitive swimmers, indicating stress to the airways (Seys et al. 2015). The absence of an effect of HME usage on cytokine concentrations may have several explanations. It could indicate that the triggers for these cytokine responses are not mediated by cooling and/or drying of the airways, or that direct sampling from the airways for local markers may be more relevant than venous sampling. Further exploration of the connection between airway and systemic inflammation resulting from exercise in cold air is warranted, as suggested by others (Bonsignore et al. 2003).

A single bout of aerobic and anaerobic exercise has been shown to induce acute increases in systemic biomarkers of oxidative stress (Bloomer and Goldfarb 2004). Oxidative stress has been detected in competitive swimmers exposed to a chlorinated environment and has also been implicated in the pathogenesis of asthma (Skrgat et al. 2018), making it a possible pathway behind the high prevalence of asthma observed in cross-country skiers (Mäki-Heikkilä et al. 2020). However, we could not detect acute increases in the oxidative stress marker 8-isoprostane concentrations in plasma or urine after exercise in the cold. Neither did HME usage significantly affect 8-isoprostane responses to exercise in cold. It is possible that the combination of triggers (temperature, humidity, exercise intensity, and duration) was insufficient, or that the time point of sampling was wrong.

### HMEs attenuate airway symptoms induced by exercise in sub-zero air

The warm-up but especially the time trial in − 15 °C induced irritation in the upper and lower airways. Irritation in the throat and chest suggests that the provocation was great enough to stimulate the parasympathetic afferent C-fiber endings below the airway epithelium. Bronchopulmonary C-fiber stimulation may by central reflex pathways and local axonal reflexes induce mucus production, cough, constriction of bronchial smooth muscle cells, extravasation, and edema of airway mucosa (Lee and Pisarri 2001). However, inhalation of the anticholinergic agent ipratropium does not appear to reduce post-exercise cough in cross-country skiers (Bordeleau et al. 2014). The usage of an HME provided good protection against these symptoms and diminished the feelings of a cold face induced by both the warm-up and time-trial exercise. That perceived thermal comfort was higher with HME is in line with our previously reported finding that skin temperature was 0.66 °C higher in men after the TT with HME, compared to without HME (Tutt et al. 2021), and suggests that a HME may prevent heat dissipation on a whole-body scale, not only from the lungs. From an athlete’s subjective user perspective, it was encouraging to see that HME usage during the exercise did not induce more dyspnea than the trial without HME. This is probably due to the low breathing resistance and the small increase in dead space for the used HME, which at high tidal volumes gives a relatively small effect on pulmonary gas fractions (Ainegren et al. 2020).

### Study limitations

The duration of the exercise was shorter than standard exercise challenge testing at room temperature (Hallstrand et al. 2018). Despite this short, intense protocol, HME usage was shown to reduce airway effects of cold-weather exercise. It remains to be clarified whether HME usage also provides protection during longer duration exercise at a lower intensity. At lower exercise intensity, the detrimental airway effects probably diminish and consequently the indication for HME usage.

The HME used in the present study slightly covered the face and significantly reduced the feeling of a cold face, which possibly prevented any bronchoconstriction induced by reduced facial cooling (Josenhans et al. 1969; Berk et al. 1987; Koskela and Tukiainen 1995; Koskela et al. 1996). Therefore, a limitation of our study is the lack of a sham trial using an HME covering the face without using a heat-exchange filter for determining the potential effect of facial cooling.

It would, of course, have been desirable to incorporate endobronchial sampling to detect any local airway cellular responses. For instance, signs of mast cell activation have been detected after broncho-provocation and exercise even in athletes without asthma or EIB (Brannan et al. 2003; Caillaud et al. 2003; Kippelen et al. 2010). Whether mast cell activation induced by exercise in cold environments could be attenuated by HMEs would require further analyses.

We do not assume that similar results will be achieved by all HMEs on the market since we have shown a large variation in breathing resistance, work of breathing, and increased dead space between different manufacturers of HMEs (Ainegren et al. 2020). The HME (Airtrim) used in the present study belongs to a group of mask-like HMEs with low breathing resistance and moderate volume (Ainegren et al. 2020). Both filter type and volume (dead space) probably affect the temperature and humidity of the inhaled air.

In another publication (Tutt et al. 2021) from the same study, we found that HME usage slightly impaired running performance (distance covered during the time trial reduced by 1.4%), but that physiological demands were similar, or in some cases, slightly higher in the HME trial. However, considering that speeds were fixed during the submaximal exercise and that the time trial was performed as a fixed duration maximal effort on each occasion, the two trials should be considered sufficiently similar to not have affected lung function per se*.* Nevertheless, the potentially small but significant negative influence on performance is an important consideration that may favor some athletes avoiding HME usage in competition. We could hypothesize that the differences in performance outcomes may be related to impaired respiratory heat loss and mild hyperthermia with the HME, as reflected by previous skin temperature and current thermal sensation results. Thus, individual consideration of the cost/benefits of HME usage should still be undertaken.

## Conclusion

This study shows that an HME that slightly covers the face, provides healthy subjects protection against acute biochemical, physiological, and symptomatic airway responses induced by warm-up followed by a 4-min maximal exercise in a very cold environment.

We find it reasonable to assume that these immediate protective effects of HMEs are transferable to other populations, irrespective of health and performance status, as long as the trigger (exercise intensity/duration, temperature, humidity) induces mild airway responses. However, whether this acute protection provides primary prevention against long-term damage and incident asthma remains to be clarified. We suggest that HME usage can be recommended as acute airway protection to healthy subjects conducting moderate-to-maximal-intensity exercise in temperatures at − 15 °C or below. However, decisions about when to use HMEs will also require consideration of their protective effects, influence on performance, and personal preferences.

## Supplementary Information

Below is the link to the electronic supplementary material.

Supplementary file1 (DOCX 14 kb)

## Data Availability

The datasets presented within are not publicly available, because consent for this was not obtained from the participants nor approved by the ethical review board. However, the data are available from the corresponding author upon reasonable request.
