# Supplementary material for: A breathing mask attenuates acute airway responses to exercise in sub-zero environment in healthy subjects
Source: Eur J Appl Physiol. 2022 Apr 7;122(6):1473–84. doi: 10.1007/s00421-022-04939-x (PMC9132816; doi:10.1007/s00421-022-04939-x)
Supplement: Supplementary file 1 — Supplementary file1 (DOCX 14 kb) [file 421_2022_4939_MOESM1_ESM.docx]

**Supplementary Table 1.** Proportion of samples within the assay range for IL-1β, GM-CSF, IL-13, IL-25 and IL-4.

|  | **HME** | | | **No HME** | | |  |
| --- | --- | --- | --- | --- | --- | --- | --- |
|  | **Pre** | **Post** | **P-value*** | **Pre** | **Post** | **P-value*** | **P-value**† |
| IL-1β | 30% | 39% | 0.365 | 22% | 30% | 0.319 | 0.365 |
| GM-CSF | 22% | 26% | 0.617 | 13% | 13% | 1.000 | 0.109 |
| IL-13 | 13% | 9% | 0.760 | 13% | 17% | 0.530 | 0.409 |
| IL-25 | 13% | 13% | 1.000 | 17% | 17% | 1.000 | 0.785 |
| IL-4 | 13% | 9% | 0.760 | 30% | 17% | 0.256 | 0.409 |

* P-value calculated for changes within trials (pre vs post)

† P-value calculated for changes between trials (proportion with HME vs proportion without HME)
